# Between Consecutive Fractures: Time and Sex as Dominant Factors in Type and Severity Concordance of Contralateral Hip Injuries

**DOI:** 10.3390/biomedicines13010114

**Published:** 2025-01-06

**Authors:** Neta Leshem, Ido Stahl, Farouk Khury, Ianiv Trior Simonovich

**Affiliations:** 1 Faculty of Medicine, Technion Israel Institute of Technology, P.O. Box 9649, Haifa 3109601, Israel; 2 Division of Orthopedic Surgery, Rambam Healthcare Campus, P.O. Box 9602, Haifa 31096, Israel; i_trior@rambam.health.gov.il (I.S.); fkhury@gmail.com (F.K.); i_stahl@rambam.health.gov.il (I.T.S.)

**Keywords:** hip fractures, classification, forecasting, fractures, multiple

## Abstract

**Background/Objectives**: Hip fractures present a global public health concern, with a forecasted rise in incidence and having associated increased mortality rates. This study aimed to investigate whether the AO Foundation/Orthopaedic Trauma Association (AO/OTA) classification of a first hip fracture can predict the location and severity of a subsequent contralateral fracture. **Methods**: We retrospectively evaluated patients with non-simultaneous bilateral hip fractures between January 2000 and February 2021 and analyzed the type and severity of each fracture using the AO/OTA classification system, interval between fractures (TI), and patients’ characteristics, including sex, age at time of each fracture, and radiographic measurements of hip morphology. **Results**: The study included 182 fractures in 91 patients (68% women, mean age: 79.5 and 82.2 years at first and second fractures, respectively; mean TI: 975 days). A strong association (lambda = 0.437, *p* < 0.001) was demonstrated between the first and second fracture classifications, which was higher in men (lambda = 0.60, *p* < 0.001) and for TI < 3 years (lambda = 0.625–0.688, *p* < 0.001). The mean TI was significantly shorter between the first and subsequent identical fractures than between different fracture types. However, mean hip morphological features did not significantly differ between groups. **Conclusions**: The initial hip fracture classification significantly predicted the type and severity of a subsequent contralateral fracture, particularly within 3 years and in men. Providing appropriate patient guidance and preventive measures is crucial, particularly for those with primary fractures that are associated with higher morbidity and mortality. Specific fracture-focused interventions, such as preventive intramedullary nail fixation, should be considered.

## 1. Introduction

Hip fractures are a global public health concern, constituting the third most common fracture type and accounting for 14% of all fractures [1]. The expected demographic changes in the upcoming decades are predicted to result in over six million hip fractures annually by 2050 [2]. Once a first hip fracture occurs, the risk for a second fracture increases [3], particularly within the first 3 years. It is common for the second fracture to occur in the contralateral hip [4]. The incidence rate for bilateral femoral fractures is estimated to be 8.54–13.8% [3,4,5,6].

Hip fractures are associated with increased mortality rates [7], with the 1-year mortality rate ranging from 14% to 36% [8]. Moreover, specific types of hip fractures have been linked to even higher mortality rates and worse functional outcomes [9]. Proximal femoral fractures are typically classified into the following two groups based on their location: extracapsular and intracapsular. The strong anatomical symmetry of the pelvis [10] suggests a possible relationship between consecutive bilateral hip fractures. Previous research reported a 71.4–91.7% prevalence of symmetric extracapsular and intracapsular fractures [3,4,11,12,13,14,15]. However, proximal femoral fractures can be further sub-categorized using various classification systems [16]. One widely accepted method for assessing fracture types is the AO Foundation/Orthopaedic Trauma Association (AO/OTA) classification system for fractures of long bones [17], which was introduced in 1996 and revised in 2018. The AO/OTA is a reliable fracture classification method based on radiographic imaging [18].

Predicting future hip fracture characteristics can aid in the implementation of targeted preventive measures, potentially reducing morbidity, mortality, and healthcare expenditure. It is proposed that once a first hip fracture occurs, a subsequent fracture would exhibit similar characteristics, including precise location, morphology, and severity, according to the AO/OTA classification system. However, to the best of our knowledge, previous studies have not assessed the combined predictive value of these characteristics. Therefore, the primary objective of this study was to evaluate the predictive value of the AO/OTA classification for subsequent contralateral hip fractures, with a secondary objective of identifying patient factors associated with recurrent fracture types.

## 2. Materials and Methods

### 2.1. Study Population

The MDClone ADAMS platform, guided by the International Classification of Diseases (ICD)-10 diagnostic coding system, was used to identify potential bilateral hip fracture cases treated at Rambam Healthcare Campus, Israel, from January 2000 to February 2021. The patient records were manually reviewed for diagnostic confirmation and study compatibility.

**Figure 1:**
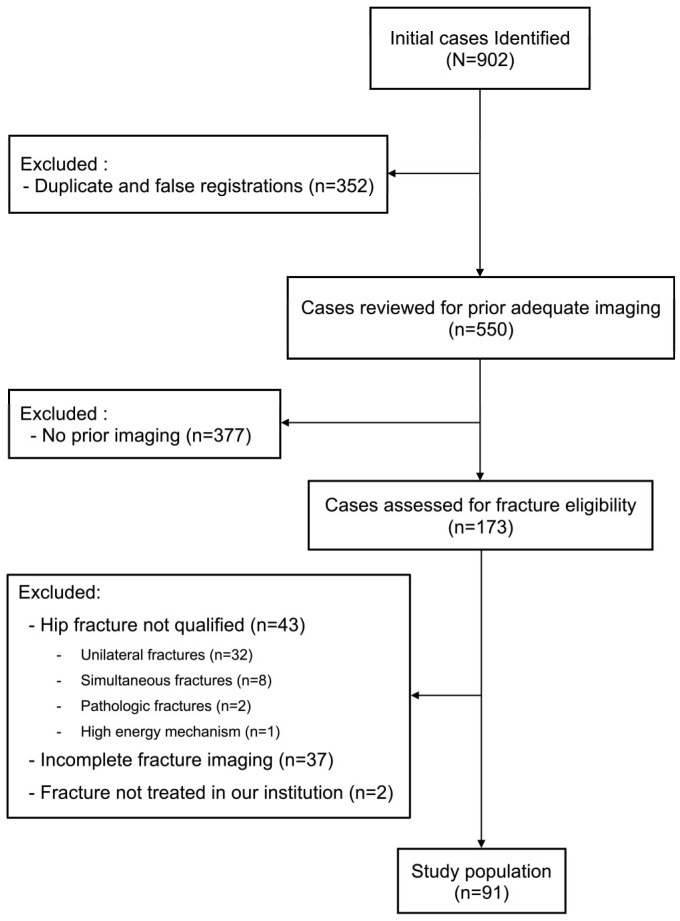
Flow chart of patient selection process.

The inclusion criteria were as follows: (1) age ≥ 55 years, (2) experienced two non-simultaneous contralateral proximal femoral fractures, (3) fracture resulting from a low-energy mechanism, (4) treated at our institution for both fractures, and (5) availability of adequate pre-first fracture hip imaging (X-ray or computed tomography (CT)). Patients with untreated fractures, fractures of pathological origin, or incomplete imaging for both femoral fractures were excluded. Figure 1 provides an overview of the eligibility criteria and the patient selection process.

Data collected from the medical records of each participant included their age at the time of both fractures and the time interval (TI) between the fractures.

### 2.2. Imaging and Measurement Tools

X-ray and CT images were selected for this study due to their widespread use as modalities, particularly in trauma cases. Patients included in this study were required to possess intact pre-injury pelvic imaging and X-ray images of each fracture (anteroposterior (AP) and lateral) captured by an experienced imaging technician (X-ray model: Presto DR, Quantum Medical Imaging, Software 2.51.02; CT model: Siemens: SOMATOM Definition Flash, 2015). All images were obtained by following a standardized radiographic protocol with proper calibration. Images of appropriate quality included no pelvic rotation or tilt, with proper femoral positioning, as previously described [19,20]. CT images were reconstructed to emulate X-ray imaging. Radiographic measurements and image adjustments were performed using PACS Version 24.2.6.5829 (Sectra Workstation IDS7, Linköping, Sweden).

### 2.3. Fracture Classification and Radiographic Measurements

Radiographs of each hip fracture case (AP and lateral) were carefully reviewed by a designated observer, who assigned the fracture type according to the AO/OTA classification [17]. This observer had been comprehensively trained in this classification method and had access to visual guides of the system.

Measurements were conducted on pre-fracture AP pelvic radiographs (48.3%, 44/91) or CT images (51.7%, 47/91) that were modified to simulate AP pelvic radiographs. The measurements of pelvic morphology were performed by a single observer who was trained in the relevant techniques and radiographic software. The assessment included measurements of both hips for each participant: alpha angle (Nötzli et al. [21]), lateral center-edge angle of Wiberg [13], acetabular inclination angle as described by Tönnis [22], acetabular (Sharp) angle [23], femoral neck shaft angle [20], hip axis length [24], femoral (neck) axis length [24], acetabular width [24], femoral neck diameter [13], and femoral head diameter [25].

The interobserver reliability was evaluated using the intraclass correlation coefficient based on readings from two independent observers, yielding a high reliability score of 0.92. To minimize bias, the observers responsible for measurements and classifications were blinded to the participants’ information. Senior orthopedic surgeons were available for consultation during the study.

### 2.4. Statistical Analysis

Statistical analyses were performed using IBM SPSS Statistics for Windows, version 25 (IBM Corp., Armonk, NY, USA). Descriptive statistics were used to measure the frequencies and prevalence of fracture types. *t*-tests were used to analyze the mean differences in age at the first and second fractures, TI between fractures, and all morphological measures in participants with identical and those with varying fractures.

The lambda coefficient [26], which measures the association between nominal variables without ordinal values, was used to assess the association between first and second fracture types. Lambda values range from 0.00 (no association) to 1.00 (perfect association), with values <0.1 indicating low association, 0.1–0.29 suggesting moderate association, and higher values indicating strong association.

The participants were categorized into four groups based on the TIs between fractures, and lambda coefficients were calculated for men, women, and all participants in each group, as well as for the entire cohort. Statistical significance was set at *p* < 0.05 for all tests.

## 3. Results

### 3.1. Study Population Demographics

An initial sample of 902 patients was retrieved from the electronic medical record database at our institution. After removing duplicate entries and false diagnostic records, 550 potentially eligible patients were identified. Further screening excluded 459 individuals (Figure 1), leaving a final cohort of 91 patients with 182 hip fractures, composed of 62 women (68.1%) and 29 men (31.9%).

Complete demographic and appropriate imaging data were available for all 91 participants. Morphological measurements were conducted on 91 pre-fracture images of the undamaged pelvis, and 364 radiographic images of the 182 fractures were used to classify the fracture types.

Table 1 presents the characteristics of the study population, including the sex distribution. The mean age at the time of the first and second fractures was comparable across sexes. However, the average TI between fractures was significantly shorter in men (398 days) than in women (*t* = 0.64, *p* < 0.001).

### 3.2. Prevalence and Distribution of Hip Fracture

**Figure 2:**
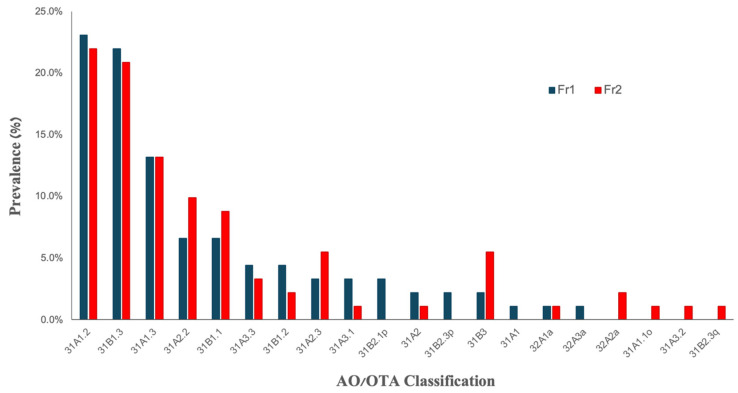
Percentage prevalence of first hip fracture (Fr1) and second hip fracture (Fr2) according to the AO/OTA classification.

In 50 patients (54.95%), the first fracture occurred in the right hip, with the most common types for both first and second fractures being 31A1.2, 31B1.3, and 31A1.3 (Figure 2). The prevalence of any other type of first fracture (FR1) was <7% and that of second fractures (FR2) was <6%. An analysis of the right and left hip fracture types showed similar distributions for the three most common fractures (Figure 2).

### 3.3. Consistency of AO/OTA Fracture Types Across Bilateral Hip Fractures

**Figure 3:**
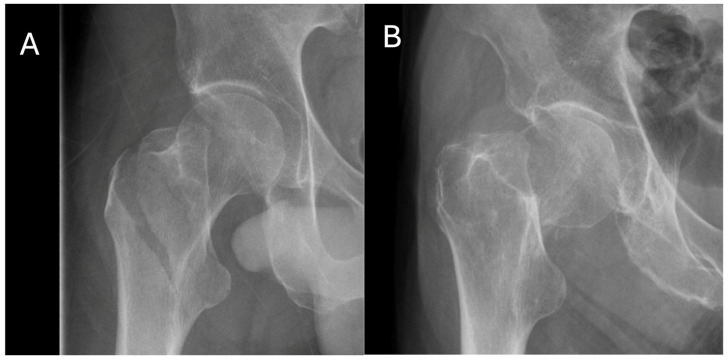
Radiographic images of the most common fracture types in the study population, according to the AO/OTA classification of femoral fractures. (**A**) 31A1.2, simple pertrochanteric two-part fracture, and (**B**) 31B1.3, femoral neck subcapital displaced fracture.

Among the 91 participants, 39 experienced identical AO/OTA fracture types in both instances. Specifically, 62% of the participants with FR1 of type 31A1.2 had the same type of FR2. Most (85.7%) patients with 31A1.2 FR1 had a 31A subtype (extracapsular) for FR2. All male patients with 31A1.2 FR1 had a 31A subtype for FR2, whereas 80% (12/15) of the female patients with this type also had a 31A subtype for their second fracture. Over half (60%) of the patients with 31B1.3 FR1 had the same type of FR2, with an additional 10% having the 31B subtype (intracapsular). All male patients with 31B1.3 FR1 had the same type of FR2, whereas 46.7% of female patients with this type had the same classification for both fractures, and 60% had any 31B subtype for their second fracture. Figure 3 provides representative images of these two fracture classifications.

### 3.4. Predictive Relationship Between Fractures

The lambda coefficient, which was used to measure the association between the patients’ first and second fracture AO/OTA types, revealed a strong correlation (lambda = 0.437, *p* < 0.001). This association was more pronounced in men (lambda = 0.60, *p* < 0.001) than in women (lambda = 0.375, *p* < 0.001). These results imply that knowledge of the FR1 type significantly improves the likelihood of accurately predicting the FR2 type, including its location and severity, by 60% and 37.5% in men and women, respectively.

### 3.5. Time Distribution for Fracture Association

To facilitate further investigation, the participants were categorized into the following four groups based on the TIs between fractures: (1) <1 year (n = 22), (2) 1–2 years (n = 26), (3) 2–3 years (n = 12), and (4) >3 years (n = 31). Table 2 presents the lambda coefficient values estimating the association between the first and second fractures across these periods for men, women, and the entire cohort. These findings indicate that the likelihood of a second fracture having the same AO/OTA classification is greatest within the first 3 years following the initial fracture. Additionally, the predictability of similar bilateral hip fractures was higher in men than women.

### 3.6. Comparative Analysis of Hip Morphology and Fracture Timing in Patients with Homogeneous vs. Heterogeneous Fracture Classifications

A comparison of the mean hip morphology factors and age at the time of fracture revealed no significant differences between patients with identical AO/OTA fracture classifications and those with different bilateral fractures (Table 3). However, the average TI between the first fracture and a similar subsequent fracture (700 days) was significantly shorter than that between different fractures (1180 days). This suggests an increased risk of a similar bilateral fracture within the first 2 years following the initial hip fracture (Table 3).

## 4. Discussion

In this study, the AO/OTA classification of hip fractures and pelvic morphology in patients with consecutive bilateral hip fractures was evaluated, confirming the hypothesis that the classification of the first fracture strongly predicts the location and severity of subsequent fractures. In addition to supporting previous studies that have demonstrated a tendency for the recurrence of fracture location [3,4,13,14,15], the current findings demonstrate a substantial predictive relationship between the location and severity of the first and second fractures. This relationship was strongest within the first 3 years following the initial fracture and was more pronounced in men. Therefore, patients with a severe initial hip fracture are likely to experience a similar severe fracture in the contralateral hip if the fracture occurs within the following 3 years.

The strong predictive value of FR1 for FR2 can potentially be explained by patient-specific features that influence the location and severity of hip fractures. Various attributes and physiological markers, such as height, bone mineral density, corticosteroid use, body fat percentage, and parathyroid hormone response to vitamin D deficiency, along with blood markers, including hemoglobin and creatinine levels, may contribute to the location and severity of proximal femur fractures [27,28]. These systemic patient-specific features are likely to exert consistent effects on fracture location and severity on both sides of the body. Furthermore, previous research has shown an association between pelvic morphology and hip fracture location [13,25]. Given the inherent symmetry of the pelvic structure [10], it is plausible that this anatomical feature could predispose individuals to similar types and severity of bilateral hip fractures.

The predictive strength of FR1 for FR2 was most pronounced within the initial 3 years post-fracture and diminished thereafter but remained significant. This is corroborated by the present finding of a longer TI between different fractures compared with that of similar fractures, which aligns with the results of previous research on contralateral hip fractures [15]. Notably, there were no significant between-group differences regarding hip morphology and age at the time of each fracture. Therefore, it is proposed that time-related changes in individual characteristics affect fracture location and severity, including alterations in hip morphology, as bones adapt to changes in mechanical loading and reduce the likelihood of similar subsequent fractures as time elapses after the initial fracture.

The lower predictive value for future fractures in women than in men may be attributed to sex-specific systemic differences. Women, who have higher osteoporosis rates, are susceptible to greater bone mass loss and structural deterioration [29,30]. Hormonal changes, particularly during menopause [31], can lead to changes in bone morphology, thereby increasing fracture risk [29,30] and influencing posture and walking patterns. These distinctive changes in women may affect the individual predictors relevant to fracture classification.

### 4.1. Clinical Implications

Predicting the classification of future hip fractures can significantly improve patient management, enabling physicians to provide more accurate risk assessments and prognoses following an initial fracture. Tailored prevention strategies can then be developed that may reduce the risk of recurring severe fractures and the associated morbidity.

Cornwall et al. [9] reported that mortality rates were the lowest in patients with non-displaced femoral neck fractures and highest in those with displaced fractures. The worst functional outcomes at 6 months were observed in patients with unstable intertrochanteric fractures. This study suggests that a second fracture will likely mirror the first fracture regarding location and severity, indicating a higher risk of recurrence in patients with initially displaced femoral neck and unstable intertrochanteric fractures. Therefore, more aggressive treatment and preventive measures are recommended for these patients, particularly men, within the first 3 years after the initial fracture.

In addition to common hip fracture prevention strategies, the ability to predict the severity of future hip fractures could prompt the consideration of new approaches. One such strategy is the preemptive fixation of the contralateral hip, similar to the procedures performed for impending fractures in metastatic disease or slipped capital femoral epiphysis. Such an approach may be particularly relevant for sub-capital hip fractures that require major surgeries, such as total or partial hip arthroplasty, because preemptive fixation can be achieved less invasively. Single-stage nailing of bilateral impending or pathological fractures has been demonstrated to be safe [32], and although it was originally developed for metastatic diseases, it can also be applied to osteoporotic fractures.

The prediction of future fracture types can also provide insight into the most likely surgical approach upon a second fracture and its associated outcomes. For example, open reduction techniques are frequently employed in complex subtrochanteric fractures and are associated with longer hospital stays, extended operative times, and increased inflammatory responses [33]. By foreseeing these factors, healthcare providers can better prepare for the treatment course and its implications, ultimately improving patient care.

### 4.2. Strengths and Limitations

The study’s demographic and statistical attributes align with those of established hip fracture research. The sex distribution of the cohort, comprising 68% women and 32% men, reflects general population trends [8,16,34] and increased risk of subsequent hip fractures in women [35,36]. The 66% 3-year cumulative incidence of bilateral fractures in this study is comparable with the 70.4% incidence reported in a large-scale study [4], suggesting the external validity and generalizability of these results to a wider bilateral hip fracture patient population. By including 13 distinct AO/OTA classifications, notably 31A1.2 and 31B1.3, the study avoids bias toward a predominant fracture type, enhancing the relevance of these insights across various hip fracture scenarios.

Nevertheless, certain limitations should be acknowledged. First, the inclusion criteria were limited to patients with non-simultaneous bilateral hip fractures treated at our institution, ensuring complete access to medical records and imaging. This could have biased our sample toward those who selected our facility for initial trauma care. Second, the prerequisite of suitable pelvic imaging excluded patients who did not undergo such imaging, resulting in an inherent selection bias. Finally, the retrospective study design limited the availability and incorporation of additional patient information such as race, osteoporosis status, body mass index, administered medications, and functional status into the analysis. Thus, it is important to note that while the results of this study are statistically significant, they should be interpreted with caution due to the limited dataset. Future studies with larger and more balanced datasets will be necessary to validate and expand upon these findings. Moreover, because our results were more significant among men, future statistical models could benefit from incorporating additional physiological markers, such as hormonal status, presence of osteoporosis, treatment history, and fall risk factors, to enhance the accuracy of predictability assessments in women.

## 5. Conclusions

This study confirms that the initial hip fracture AO/OTA classification strongly predicts the location and severity of a subsequent contralateral fracture, particularly within the first 3 years and more prominently in men. Patient-specific features that exert similar effects on both sides of the body, such as bone mineral density, blood markers, and hip morphology, can potentially explain this relationship. These findings underscore the importance of tailored prevention strategies and suggest the potential of preemptive contralateral hip fixation for mitigating the risk of severe recurrent fractures in high-risk patients.

## Figures and Tables

**Table 1:** Characteristics of study population including *t*-test for sex mean differences.

Characteristics	Full Sample (n = 91) Mean ± SD	Women (n = 62) Mean ± SD	Men (n = 29) Mean ± SD	*p*-Value ^†^
Age at time of first fracture (years)	79.5 ± 9.3	79.6 ± 9.1	79.4 ± 9.8	0.92
Age at time of second fracture (years)	82.2 ± 9.7	83.6 ± 9.7	81.3 ± 9.9	0.56
Time interval between fractures (days)	975 ± 897	1102 ± 994	704 ± 569	0.02

SD, standard deviation. ^†^ Independent-samples *t*-test analyses.

**Table 2:** Lambda test for association between first and second fracture over time with sex-based distribution.

Fracture Time Interval	Population	Lambda	*p*-Value
<1 Year	Male	1.000	<0.0001
	Female	0.556	0.005
	Total	0.688	<0.0001
1–2 Years	Male	1.000	0.007
	Female	0.600	0.013
	Total	0.625	<0.0001
2–3 Years	Male	1.000	0.07
	Female	0.800	0.002
	Total	0.667	0.001
>3 Years	Male	0.800	0.002
	Female	0.278	0.11
	Total	0.360	0.026

**Table 3:** Comparison of hip morphology, fracture timing, and age between identical and different fractures.

Morphologic Characteristics	Identical Fractures (n = 39)		Different Fracture (n = 52)		*p*-Value ^†^
	Mean	SD	Mean	SD	
Alpha angle (left)	47.6	6.48	50.18	11.19	0.17
Alpha angle (right)	46.1	6.99	49.8	13.09	0.09
LCEA (left)	30.46	5.63	32.12	7.47	0.23
LCEA (right)	29.76	5.76	32.74	7.72	0.038
Acetabular angle (left)	38.71	3.61	39	3.45	0.7
Acetabular angle (right)	38.73	3.65	37.6	3.66	0.15
Acetabular index (Tönnis) (left)	6.97	3.68	6.21	4.03	0.35
Acetabular index (Tönnis) (right)	5.53	3.05	4.86	4.04	0.37
Neck–shaft angle (left)	131.5	5.51	129.51	5.95	0.10
Neck–shaft angle (right)	130.89	6.89	130.27	4.74	0.63
Hip axis length (left)	118.4	13.87	116.75	11.53	0.55
Hip axis length (right)	118.06	13.89	116.97	11.35	0.69
Femoral axis length (left)	100.36	10.12	100.39	9.2	0.99
Femoral axis length (right)	100.43	9.77	100.43	9.29	0.99
Acetabular width (left)	18.04	5.06	16.36	4.51	0.10
Acetabular width (right)	17.63	5.07	16.54	4.25	0.28
Femoral neck diameter (left)	34.08	3.61	34.44	3.44	0.63
Femoral neck diameter (right)	33.59	3.26	34.26	3.39	0.35
Femoral head diameter (left)	48.73	4.46	48.65	4.9	0.93
Femoral head diameter (right)	49.03	4.24	49.07	3.71	0.96
Age (1st fracture)	80.6	10.14	78.76	8.54	0.36
Age (2nd fracture)	82.51	10.57	82	9.15	0.81
TI (days)	700.44	625.12	1180.77	1014.2	0.007

LCEA, lateral central edge angle; TI, time interval; SD, standard deviation. Notes: Lengths were measured in millimeters (mm) and angles in degrees (°). ^†^ Independent-sample *t*-test.

## Data Availability

The raw data supporting the conclusions of this article will be made available by the authors on request.
